# HBV preS2 promotes the expression of TAZ via miRNA-338-3p to enhance the tumorigenesis of hepatocellular carcinoma

**DOI:** 10.18632/oncotarget.4804

**Published:** 2015-08-05

**Authors:** Peng Liu, Hualin Zhang, Xiaohong Liang, Hongxin Ma, Fang Luan, Bo Wang, Fuxiang Bai, Lifen Gao, Chunhong Ma

**Affiliations:** ^1^ Key Laboratory for Experimental Teratology of Ministry of Education and Department of Immunology, Shandong University School of Medicine, Jinan, Shandong, 250012, P.R. China; ^2^ Department of Clinical Laboratory, Shandong Provincial Hospital affiliated to Shandong University, Jinan, Shandong, 250021, P.R. China

**Keywords:** preS2, miRNA-338-3p, HCC, Hippo pathway, TAZ

## Abstract

Transactivators encoded by HBV, including HBx and preS2, play critical role in hepatocellular carcinoma (HCC). YAP, a downstream effector of the Hippo pathway, is involved in hepatocarcinogenesis mediated by HBx. Here, we investigated whether preS2, another transactivator encoded by HBV, regulates the Hippo pathway to promote HCC. We found that preS2 overexpression upregulated TAZ, a downstream effector of the Hippo pathway, at protein level but not at mRNA level. preS2 suppressed miRNA-338-3p expression in HCC cell lines. miRNA-338-3p mimics downregulated TAZ, while miRNA-338-3p inhibitor restored the expression of TAZ, suggesting that TAZ is a direct target of miRNA-338-3p. TAZ overexpression stimulated growth of HCC cell lines. Knockdown of TAZ dampened preS2-promoted HCC proliferation and migration. Thus, preS2 upregulates TAZ expression by repressing miRNA-338-3p. TAZ is necessary for preS2-promoted HCC proliferation and migration

## INTRODUCTION

Hepatitis B virus (HBV) infection is the most serious and prevalent chronic viral infection in the world [1]. It is estimated that more than 2 billion people have been infected by HBV, and more than 350 million are chronic carriers of the virus [1, 2]. People infected with HBV are more susceptible to develop hepatocellular carcinoma (HCC) [2], which is the third leading cause of cancer related death in the world [3]. In fact, about half of the total liver cancer mortality was attributed to HBV infection [4].

It has been well documented that HBV-DNA integrated into the host genome in almost all the HBV related HCC, leading to the expression of viral proteins [5, 6]. Among the four functional proteins encoded by HBV(X, surface, core, and polymerase), the C-terminal truncated middle surface protein (MHBs^t^) was found to be integrated into 1/3 of the host genome in HBV related HCC [7, 8]. The preS2 domain of MHBs^t^ was identified to be the minimal functional fragment [9, 10]. Our previous study showed that preS2 transactivate oncogenes, including hTERT and Foxp3, to promote the progression of HCC [11, 12]. However, the detailed molecular mechanism of preS2 in the promotion of HCC is still mainly unknown.

The Hippo pathway, first discovered in *Drosophila* in 2003, is critical in controlling organ size by regulating both cell proliferation and apoptosis [13–16]. Transcription co-activators Yes-associated protein (YAP) and transcriptional co-activator with a PDZ binding domain (TAZ) are the major downstream effectors of Hippo pathway [17–19]. Accumulated data demonstrate that dysregulation of Hippo pathway is actively involved in tumorigenesis [17, 18, 20–22]. As a key transducer of Hippo pathway, TAZ has been demonstrated as oncogene in many cancers, including breast cancer, non-small cell lung cancer, etc [23, 24]. TAZ not only promotes the proliferation and epithelial-mesenchymal transition of cancer cells [25, 26], but also confers cancer stem cell-related traits on the cancer cell [27]. Zhang recently reported that HBx enhances the expression of YAP by CREB to promote HCC progression [28]. However, whether HBV could regulate TAZ to promote the progression of HCC is completely unknown.

In the present study, we showed evidence that preS2 upregulated TAZ expression by modulating miRNA-338-3p. In addition, the role of TAZ in preS2 mediated HCC proliferation and migration of HCC was evaluated *in vitro*.

## RESULTS

### preS2 posttranscriptionally upregulates TAZ expression

**Figure 1:**
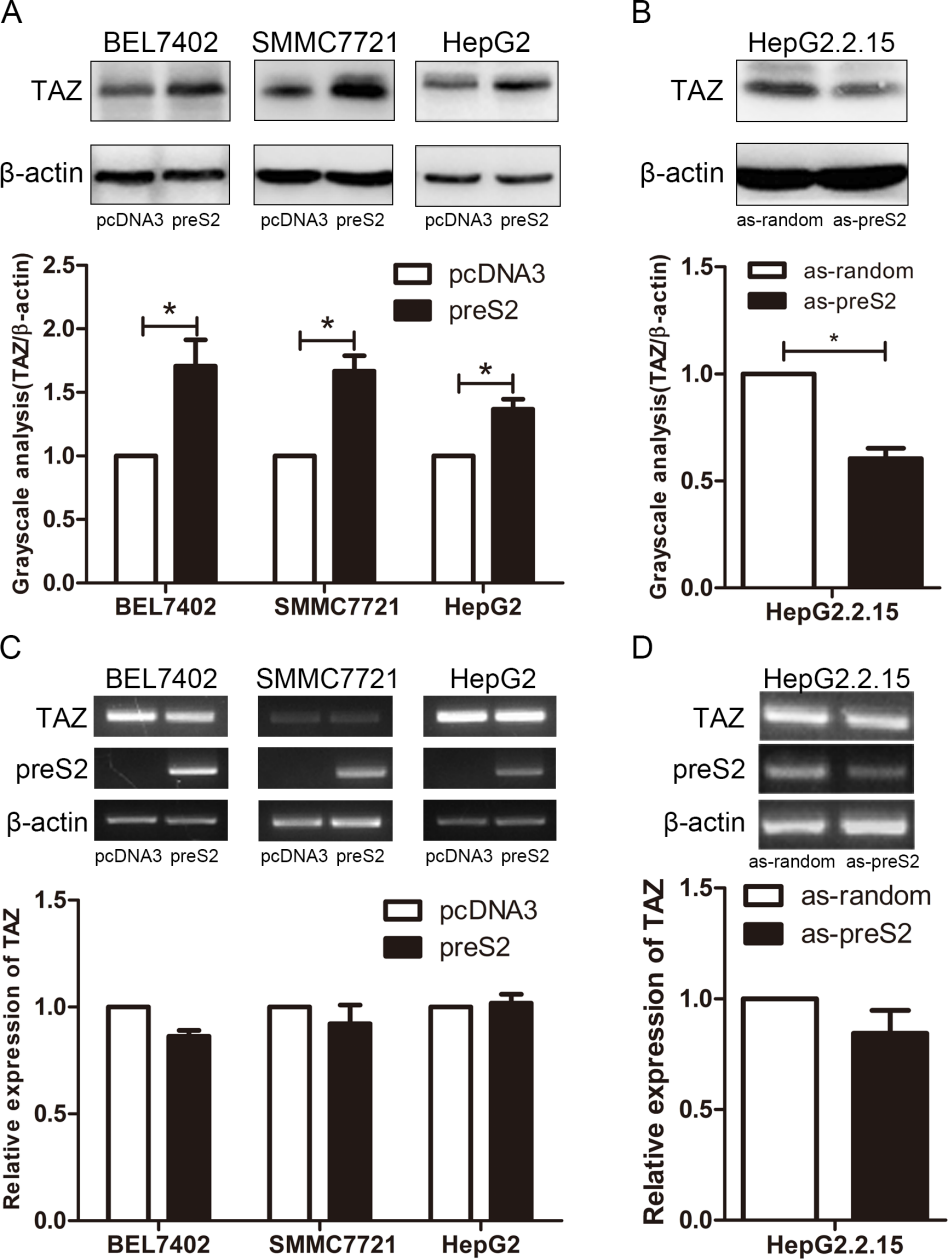
preS2 upregulates TAZ expression at the protein level **A.** BEL7402, SMMC7721 and HepG2 cells were transfected with pcDNA3 or preS2 expressing plasmid respectively. Western blot was applied to detect TAZ expression 48 h after transfection. **B.** HepG2.2.15 was transfected with oligo as-random or as-preS2. TAZ protein expression was determined 48 h after transfection. **C, D.** TAZ mRNA was detected by RT-PCR (up panel) or qRT-PCR (down panel) in transfected HCC cells described in (A) and (B) *p < 0.05.

HBV infection is a risk factor for HCC and HBx has been well known involved in this process. In addition, accumulated evidence confirm the role of preS2 in promoting hepatocarcinogenesis by activating oncogenes or pathways, such as hTERT and c-raf-1/Erk2 [8, 12]. Here, we tried to explore whether HBV could activate TAZ expression in HCC cells. Firstly, we compared the endogenous expression of TAZ in different HCC cell lines by PCR and western blot. Very interestingly, compared with its parent cell line HepG2, HepG2.2.15 cells which harbor 4 copies of HBV DNA showed relative higher level of TAZ (Supplementary Figure S1), indicating a positive correlation between HBV and TAZ expression. To further evaluate the regulation of HBV encoding proteins on TAZ, both overexpression and knockdown assays were performed in HCC cell lines. As shown in Figure 1, preS2 overexpression significantly enhanced TAZ protein level in all detected HCC cell lines (Figure 1A), and knockdown of preS2 inhibited TAZ expression (Figure 1B). However, TAZ mRNA was not significantly affected in these cells (Figure 1C and 1D). These results suggested that preS2 could posttranscriptionally upregulate TAZ expression in HCC cells. Interestingly, our data also showed that HBx increased the protein level but not the mRNA level of TAZ in different HCC cell lines (Supplementary Figure S2), indicating that not only preS2 but also HBx could enhance TAZ expression.

### preS2 inhibits the expression of miRNA-338-3p

**Figure 2:**
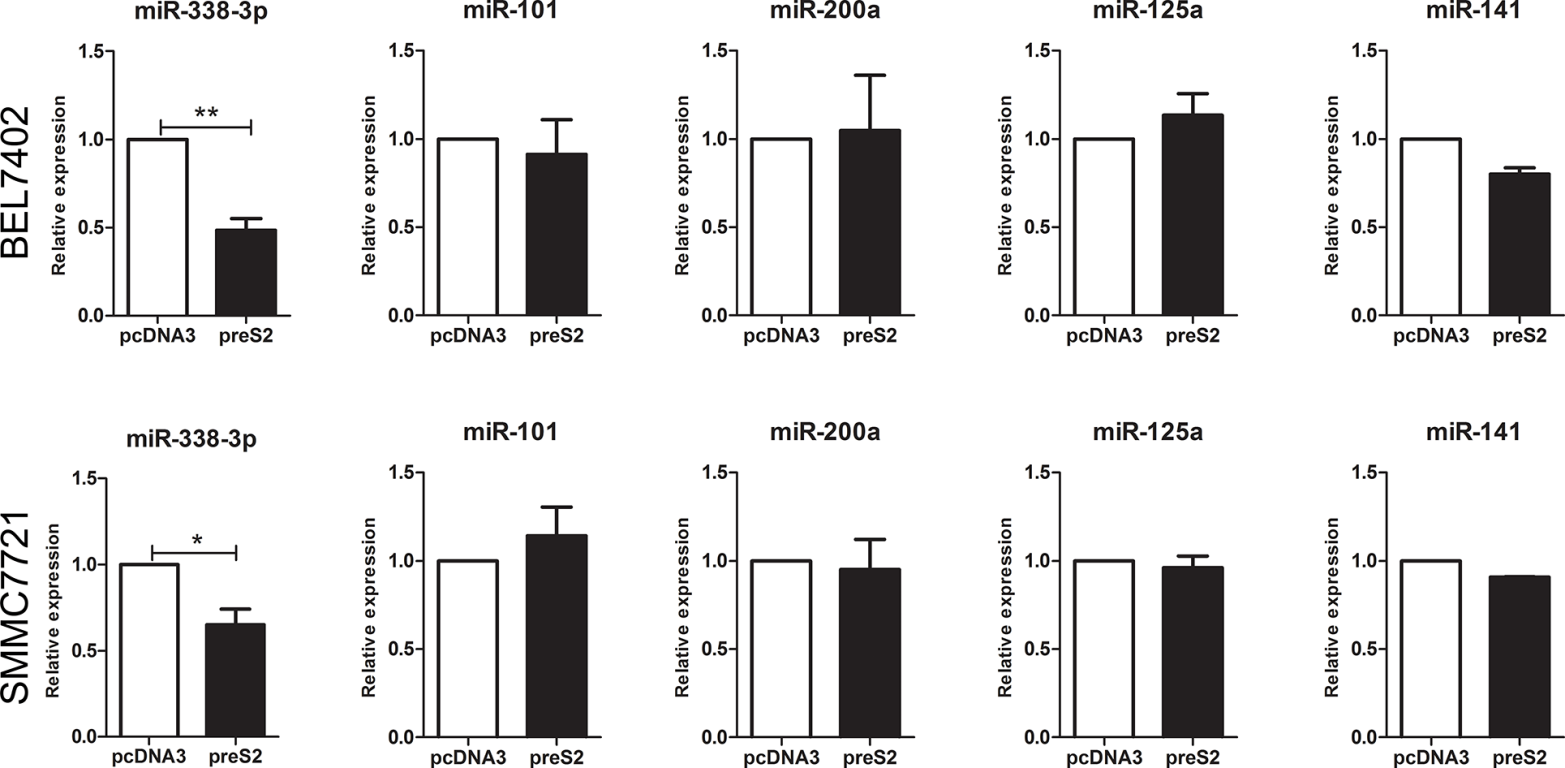
preS2 downregulates the expression of miRNA-338-3p qRT-PCR analysis of indicated miRNAs in BEL7402 and SMMC7721 cells transfected with pcDNA3 or preS2. U6 was used as an internal control. This experiment was repeated 3 times and data was shown as mean ± SD. *p < 0.05, **p < 0.01.

It is well documented that HBV is actively involved in the development of HCC by modulating miRNAs [29-31], which regulate target gene expression at the posttranscriptional level. Thus, we came to evaluate whether preS2 could regulate TAZ expression by modulating miRNAs. Several miRNAs were predicted to target TAZ by miRNA computational target prediction tools including TargetScan, miRanda and PicTar (Supplementary Table S1). Based on their previously reported roles in HCC [32-35] and the proved repression effect on TAZ [36, 37], five miRNAs, including miR-101, miR-338-3p, miR-200a, miR-125a and miR-141, were selected for further investigation. In order to test whether preS2 regulate these miRNAs' expression, preS2 was transfected into HCC cell lines and qRT-PCR was involved to assay the miRNA expression. As shown in Figure 2, only the expression of miR-338-3p but not the other miRNAs was inhibited in preS2 overexpressing BEL7402 and SMMC7721 cells. These results indicated that preS2 inhibited miRNA-338-3p expression.

### TAZ is a cellular target of miRNA-338-3p

**Figure 3:**
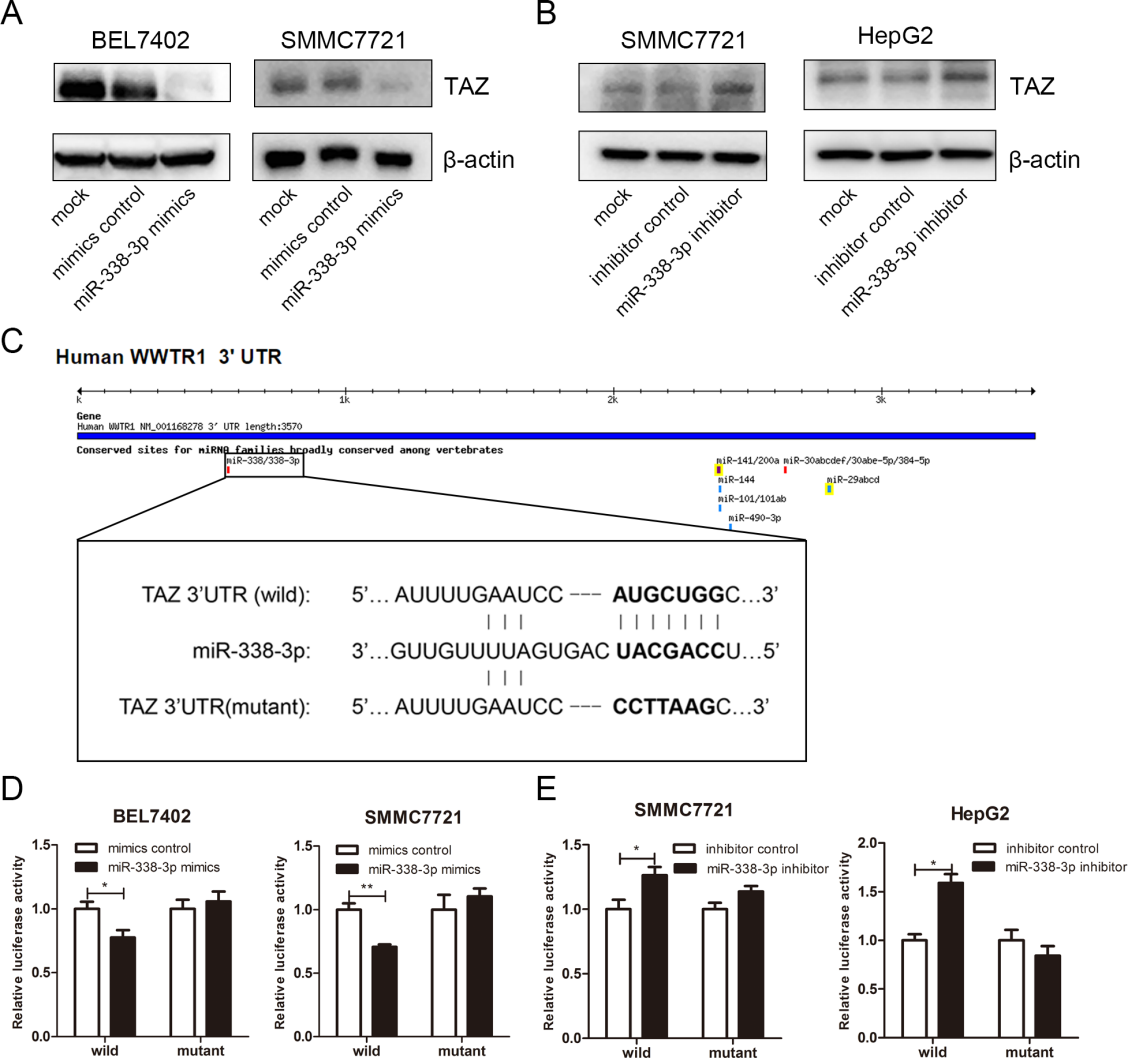
TAZ is a target gene of miRNA-338-3p **A, B.** HCC cell lines were transfected with miRNA-338-3p mimics and mimics control (A) or miRNA338-3p inhibitor and its corresponding negative control (B) The protein level of TAZ was determined by western blot. β-actin served as the internal control. **C.** Schematic of the reporter plasmids containing wild or mutant 3′UTR of TAZ. The complementary site of the seed sequence of miRNA-338-3p was selected for mutation. **D, E.** HCC cell lines were transfected with miRNA-338-3p mimics and its negative control (D) or miRNA-338-3p inhibitor and its negative control (E) Luciferase activity was detected 48 h after transfection. Renilla activity served as transfection control. The experiments were repeated for 3 times and data was shown as mean ± SD. *p < 0.05, **p < 0.01.

We next evaluated whether TAZ is a cellular target gene of miR-338-3p. First, the endogenous expression of miR-338-3p was detected in the HCC cell lines. Interestingly, compared with HepG2 cells which showed relative low protein level of TAZ and high level of miR-338-3p, HepG2.2.15 cells with higher endogenous expression of TAZ displayed very little miR-338-3p expression (Supplementary Figure S3), indicating the negative correlation between miR-338-3p and TAZ. In order to verify the regulation of TAZ by miRNA-338-p, both miR-338-3p mimics and inhibitors were transfected into HCC cell lines, the mock cells and cells transfected with control oligos of mimics or inhibitor were used as control. As shown in Figure 3, miRNA-338-3p mimics inhibited TAZ protein expression in BEL7402 and SMMC7721 cells (Figure 3A). Moreover, miR-338-3p mediated repression on TAZ could be acquired in HBV-replicating HepG2.2.15 cells (Supplementary Figure S4), consistent with the hypothesis that miR-338-3p repressed TAZ expression under the circumstance of HBV infection. On the other hand, miRNA-338-3p inhibitor greatly increased TAZ protein level in SMMC7721 and HepG2 cells (Figure 3B). However, qRT-PCR assay did not display any significant changes of TAZ mRNA level between miRNA-338-3p overexpressed HCC cells and control cells (Supplementary Figure S5). All these results suggested that miRNA-338-3p downregulated TAZ expression at posttranscriptional level, which is consistent with the effect of preS2 on TAZ expression (Figure 1A).

To further confirm TAZ as the direct and specific target of miRNA-338-3p, luciferase reporter plasmids containing *TAZ* 3′UTR with the potential binding site of miRNA-338-3p (*TAZ* 3′UTR-wild) or *TAZ* 3′UTR with mutation at miRNA-338-3p binding site (*TAZ* 3′UTR-mutant) were prepared and involved in the luciferase assay (Figure 3C). As shown in Figure 3D, miRNA-338-3p mimics decreased the reporter activity of *TAZ* 3′UTR-wild but not *TAZ* 3′UTR-mutant in BEL7402 and SMMC7721 cells. On the other hand, miRNA-338-3p inhibitor increased the reporter activity of *TAZ* 3′UTR-wild in HCC cells, but not the reporter activity of *TAZ* 3′UTR-mutant (Figure 3E). Taken together, these results suggested that TAZ is a potential target gene of miRNA-338-3p.

miR-338-3p has been identified as a negative regulator in HCC by down regulating different target genes including cyclin D1 and smoothened [34, 38]. In order to verify the role of miR-338-3p in repression of TAZ *in vivo*, H22 homografts were prepared in Balb/c mice. As expected, overexpression of miR-338-3p inhibited the tumor growth *in vivo* (Supplementary Figure S6). Accompanied with the forced expression of miR-338-3p, the TAZ expression was significantly reduced (Supplementary Figure S6E).

### preS2 upregulates TAZ via miR-338-3p

**Figure 4:**
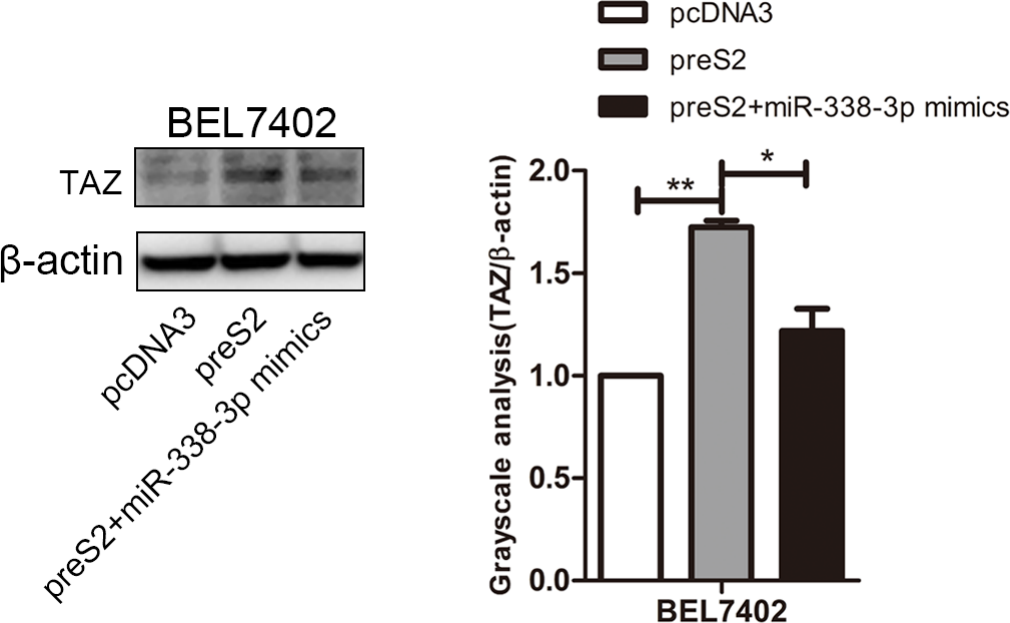
preS2 promoted TAZ expression via miR-338-3p BEL7402 cells were transfected with pcDNA3, preS2 or preS2 plus miR-338-3p mimics. The expression of TAZ was detected by western blot 48 h after transfection and β-actin served as the internal control.

To further confirm the effect of miR-338-3p in the process of preS2-mediated TAZ overexpression, the rescue experiment was conducted. As shown in Figure 4, preS2 overexpression significantly upregulated TAZ expression in BEL7402 cells and co-transfection of miR-338-3p mimics with preS2 greatly dampened preS2-mediated upregulation of TAZ. This result clearly supported the hypothesis that miR-338-3p is one of the powerful mechanisms of preS2 mediated upregulation of TAZ.

### TAZ promotes the growth of HCC cell lines

**Figure 5:**
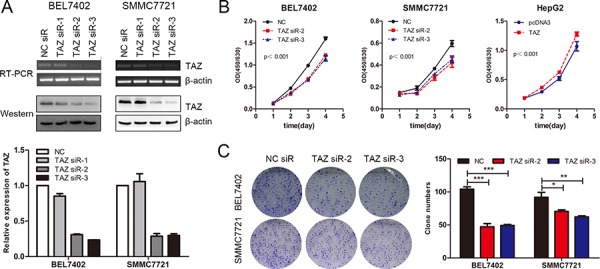
TAZ is involved in the progression of HCC **A.** TAZ siRNAs were transfected into SMMC7721 and BEL7402 cells and the TAZ expression was determined by RT-PCR, western blot and qRT-PCR (down panel) respectively. β-actin was used as the internal control. **B.** BEL7402 and SMMC7721 cells were transfected with TAZ siRNAs while HepG2 cells were transfected with TAZ expressing plasmid. The proliferation rate as determined by CCK-8 assay. **C.** Colony formation assay was performed in BEL7402 and SMMC7721 cells transfected with TAZ siRNAs. The experiments were repeated 3 times and data was shown as mean ± SD. One of the representative colony formation assay was also shown. *p < 0.05, **p < 0.01, ***p < .001.

TAZ has been reported to promote the progression of breast cancer and non-small cell lung cancer [23, 25]. To evaluate the role of TAZ in HCC, TAZ knockdown assays were performed in BEL7402 and SMMC7721 cells with comparable high level of endogenous TAZ expression. Results of PCR and western blot demonstrated that TAZ was efficiently silenced by TAZ siRNA (siR-2 and siR-3) (Figure 5A). Accompanied with the knockdown of TAZ, cell growth of BEL7402 and SMMC7721 cells was decreased. On the other hand, overexpression of TAZ in HepG2 cells promoted the cell proliferation (Figure 5B), suggesting that TAZ promoted the cell growth of HCC cell lines. This result is further supported by the colony formation assay. As shown in Figure 5C, TAZ knockdown significantly repressed the colony formation ability of BEL7402 and SMMC7721 cells. Taken together, these results indicated that TAZ promoted the growth of HCC cell lines, which is consistent with the reported oncogenic role of TAZ in HCC and many other tumors [23, 25, 39].

### preS2 enhances the proliferation of HCC cells by TAZ *in vitro*

**Figure 6:**
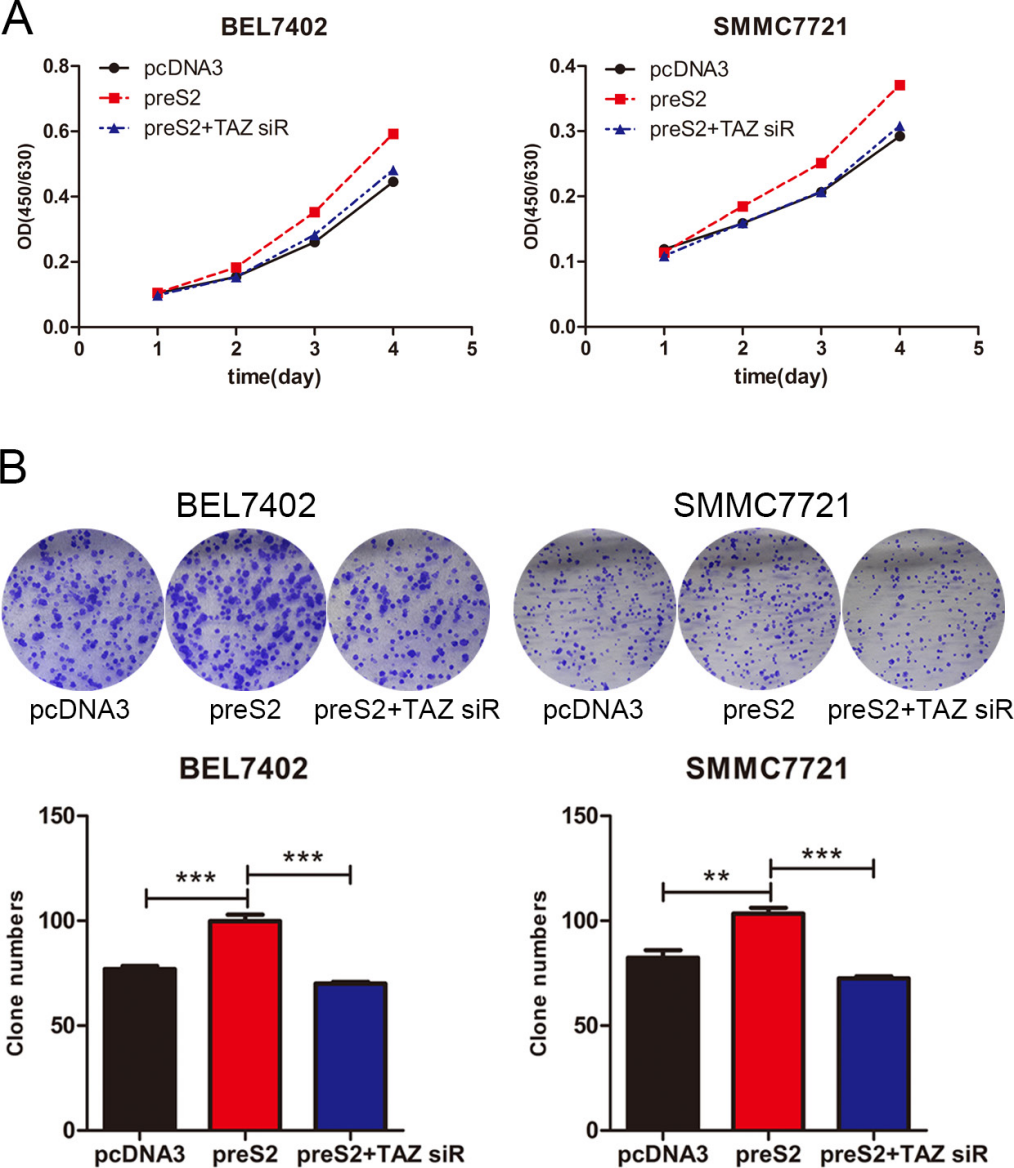
preS2 promotes the proliferation of HCC by TAZ **A.** The proliferation rate of BEL7402 and SMMC7721 transfected with pcDNA3, preS2 or preS2 plus TAZ siRNA were determined by CCK-8 assay. **B.** The colony formation ability of BEL7402 and SMMC7721 transfected with pcDNA3, preS2 or preS2 plus TAZ siRNA were determined. The experiments were repeated 3 times and data was shown as mean ± SD. One of the representative colony formation assay was also shown. *p < 0.05, **p < 0.01, ***p < .001.

We previously showed that preS2 could promote the tumorigenesis of HCC partially by activating *hTERT* and *FOXP3* expression [11, 12]. Here we estimated whether preS2 could promote the proliferation of HCC by regulating *TAZ*. To address this, BEL7402 and SMMC7721 cells were co-transfected with TAZ siRNA and preS2 expressing plasmid. The proliferation rate was determined by CCK-8 assay. As shown in Figure 6A, preS2 promoted the HCC cells proliferation, while TAZ siRNA almost completely impaired preS2-enhanced cell growth in these cells. As expected, similar results were acquired in the colony formation assay. preS2 overexpression exacerbated the colony formation of BEL7402 and SMMC7721 cells and this effect was impaired by TAZ siRNA (Figure 6B). These results suggested that preS2 promotes HCC cells proliferation by activating TAZ.

### preS2 accelerates the migration of HCC cells via TAZ *in vitro*

**Figure 7:**
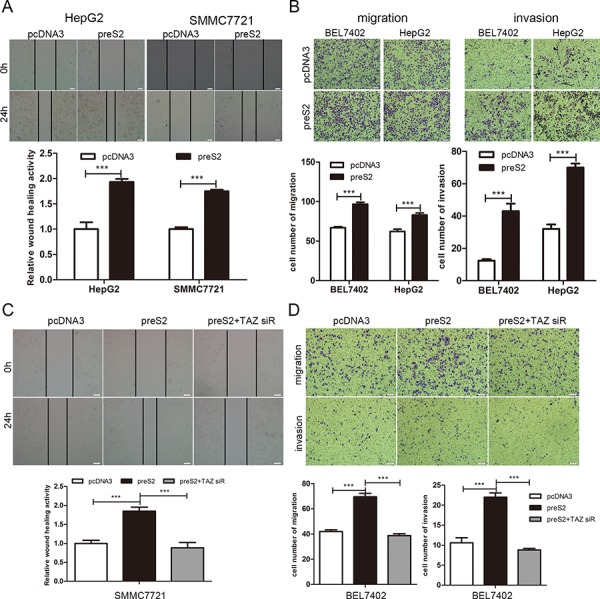
preS2 promotes the migration of HCC by TAZ **A, B.** HCC cells were transfected with pcDNA3 or preS2. Wound healing (A) and migration or invasion ability (B) was determined at the indicated times. **C, D.** HCC cells were transfected with pcDNA3, preS2 or preS2 plus TAZ siRNA. Wound healing (C) and migration or invasion ability (D) was determined at the indicated times. The experiments were repeated 3 times and data was shown as one of the representative. *p < 0.05, **p < 0.01, ***p < .001.

It has been reported that TAZ is involved in the metastasis of solid tumors [40, 41]. This prompted us to investigate whether preS2 could accelerate the HCC cell migration by TAZ. Thus, both wound healing and transwell assay were conducted. Results of wound healing assays showed that, preS2 overexpression greatly promoted the cell motility of HepG2 and SMMC7721 cells (Figure 7A), while knockdown of preS2 inhibited HepG2.2.15 cells' wound healing ability (Supplementary Figure S7). Consistently, transwell assay also showed that preS2 promoted the migration and invasion ability of HCC cells (Figure 7B). In order to estimate the involvement of TAZ in preS2-mediated effects on HCC cell motility, rescue assays were performed. As shown in Figure 7C, co-transfection of preS2 with TAZ siRNA significantly repressed preS2-enhanced cell motility. Similar results were obtained from the migration and invasion assays. preS2 overexpression increased the migration and invasion ability of BEL7402 cells and this effect was impaired by TAZ siRNA (Figure 7D). These results suggested that preS2 promoted the migration of HCC by activating TAZ.

## DISCUSSION

Although the molecular mechanism of HCC progression is complicated and still not well established, the environmental factors, especially the hepatitis virus infection are believed to increase the risk of HCC development. It has been reported that HBsAg carriers have 25-37 times increased risk of HCC compared with the non-infected individuals [42, 43]. HBV encoded protein regulates host cell gene expression or signal pathway to promote the hepatocarcinogenesis. In this study, we demonstrated for the first time that preS2/miRNA-338-3p/TAZ pathway regulates the growth and migration of HCC cells *in vitro*. Our results offer a new perspective in understanding the pathology mechanism of HBV-associated HCC.

Hippo pathway has been recently identified as a critical regulator in tumorigenesis, especially in hepatocarcinogenesis [20]. Accumulated data demonstrated the important oncogenic role of YAP, one of the key downstream effector of the Hippo pathway, in HCC development [20, 28]. However, study on TAZ, another key transcription co-activator of the Hippo pathway in HCC is limited. Here, we provide evidence to show that preS2 promote HCC proliferation and migration via upregulating TAZ expression. As reported in many other tumors [22, 25], our results showed that TAZ not only enhanced cell growth but also promoted the colony formation of cultured HCC cell lines (Figure 5). This is consistent with two papers which were just published during our preparation of the manuscript [39, 44]. They both reported TAZ was upregulated in liver tumor tissues compared with that in adjacent non-tumorous tissues. Furthermore, TAZ knockdown results in inhibited cell proliferation and migration of cultured HCC cells, therefore effectively inhibits the tumor growth and metastasis of transplanted liver tumors in mice [39]. Our data further suggested that TAZ is necessary for preS2 mediated HCC proliferation and migration. Knockdown of TAZ in preS2-overexpessing cells significantly suppressed both the cell growth and cell migration of detected HCC cell lines (Figure 6 and Figure 7). Collectively, our and previous study suggested that TAZ is involved in the development of HBV related HCC. Given that TAZ does not bind the genome DNA directly, the molecular mechanism by which TAZ is involved in the progression of HCC should be further investigated.

preS2 is the minimal functional domain of MHBs^t^ encoded by integrated HBV S gene which accounts for more than one-third of HBV integration in HBV-associated HCC [9, 10, 45]. It has been well studied that preS2 works as a promoter of HCC by activating oncogenes, including hTERT and Foxp3 [11, 12] or triggering the activation of tumor-promoting signaling [8]. Here, we showed evidence supporting that preS2 enhanced protein level of TAZ in HCC cells by repressing miRNA-338-3p expression, which is implicated in hepatocarcinogenesis [34, 46]. preS2 overexpression significantly suppressed the expression of miRNA-338-3p in BEL7402 and SMMC7721 cells (Figure 2). As far as we know, this is the first report identifying microRNA as target of preS2. However, the mechanism by which preS2 modulates the expression of miRNA-338-3p remains to be investigated. It will also be interesting to examine whether preS2 could regulate the expression of other miRNAs.

Dysregulation of miRNA expression is a character of tumor. Individual miRNA can have tumor-suppressive or tumor-promoting functions [47]. Previous studies suggested the close correlation between Hippo pathway and microRNA. Hippo pathway mediated the widespread suppression of microRNA in human cancers [48, 49]. Conversely, different components of Hippo pathway could be regulated by microRNA. The upstream molecule *Mst1* and *Mst2* could be targeted by miRNA-138 and miRNA-133b respectively [50, 51]. *Lats2* could be repressed by miRNA-31, miRNA-93, and miRNA-135b [52–54]. Moreover, the effective molecule *YAP* is repressed by miRNA-375, miRNA-141 andmiRNA-200a [55–57]. In the present study, we demonstrated that miRNA-338-3p repressed the expression of *TAZ*. miRNA-338-3p mimics downregulated the expression of TAZ while miRNA-338-3p inhibitor restored the expression of TAZ. Luciferase assay further suggested that miRNA-338-3p targets the 3′UTR of *TAZ* (Figure 3D and 3E). During the preparation of our manuscript, it was reported that TAZ could be targeted by miR-125a in glioblastoma and miR-141 in gastric cancer respectively [36, 37], however, whether TAZ could be regulated by microRNA in HCC was not elucidated. Meanwhile, in our experiments, the expression of miR-125a and miR-141 was not regulated by preS2, indicating that these two miRNA were not involved in preS2-induced TAZ overexpression. These strongly suggested that TAZ might be regulated by different miRNA in different tissues. In another word, TAZ regulation has tissue specific mechanisms.

In summary, our data suggested that TAZ was involved in the growth of HCC cells. preS2 upregulated the expression of TAZ by modulating miRNA-338-3p to promote the progression of HCC. The preS2/miRNA-338-3p/TAZ pathway might be an important driver of the development of HBV related HCC. Our findings provide new insights into virus mediated hepatocarcinogenesis.

## MATERIALS AND METHODS

### Computational target prediction and luciferase activity assay

Computational target prediction tools, including TargetScan (http://www.targetscan.org), PicTar (http://www.pictar.mdc-berlin.de) and miRanda (http://www.microrna.org) were applied to assess the miRNAs potential targeting TAZ. The human TAZ-3′UTR sequence was cloned into pGL3-promoter vector (Promega, Madison, WI, USA) to get pGL3-promoter-TAZ-3′UTR (wild). Another pGL3-promoter luciferase plasmid containing TAZ-3′UTR sequence with a mutation in the putative miR-338-3p seed sequence was also generated and named as pGL3-promoter-TAZ-3′UTR (mutant). HCC cells were plated in 48-well plates and allowed to reach 80% confluence before being transfected with the reporter construct and miR-338-3p mimics or inhibitor. Cells were harvested 48 hours after the transfection and luciferase activity was measured using the Dual-Luciferase Reporter Assay System (Promega, Madison, WI, USA) according to the manufacturer's recommendations. Data was normalized for transfection efficiency by division of firefly luciferase activity with that of Renilla.

### Cell culture and transfection

The human HCC cell lines BEL7402, SMMC7721 were cultured in RPMI 1640 medium. HepG2 cells were cultured in minimum essential medium with sodium pyruvate, and HepG2.2.15 was cultured in minimum essential medium with 380 ng/ml G418 (Sigma). All the cells were purchased from Shanghai Institute of Cell Biology, Chinese Academy of Sciences (Shanghai, China), and all the medium was supplemented with 10% fetal bovine serum (Gibico). Murine liver cancer cell line H22 was passaged in mouse peritoneal cavity and freshly collected before each experiment. For transient transfection of plasmids into HCC cell line, Lipofectamine™ 2000 (Invitrogen, USA) was used as the manufacturer's instruction. For transient silencing, siRNA was transfected into cells with the GenePORTER2 transfection reagent (Genlantis, San Diego, CA) according to the protocol.

### Plasmids and siRNAs

Human TAZ expression plasmid pcDNA3-TAZ was generated by cloning the cDNA amplified with PCR into pcDNA3 (Invitrogen, Grand Island, NY, USA). Expression plasmid pcDNA-preS2 which encodes HBV preS2 gene fused with HA-tag was described previously [58]. To block preS2 expression in HepG2.2.15, oligo nucleotide complementary to preS2 (5′CCACTGCATGGCCTGAG3′), designated as as-preS2 was synthesized by Biosune Corporation (Shanghai, China). As a control, a random 15 mer oligonucleotide (5′TTGCCGAGCGGGGTA3′), unrelated to HBV genome and any genomic sequence, designated as as-random was also synthesized simultaneously. Human TAZ siRNA was designed and synthesized by Genepharma (Shanghai, China) and the siRNA sequences were shown in Supplementary Table S3.

### Cell growth curve and colony formation assay

Cell viability was measured with the Cell Counting Kit-8 (Dojindo, Shanghai, China) according to the manufacturer's instruction. Standard colony formation assays were performed as described previously [12]. Briefly, transfected cell were plated in 6-well plates at the density of 1500 cell per well. Ten days later, colonies were fixed by methanol and stained by crystal violet for 20 minutes. Each experiment was repeated at least three times.

### Western blot

Cells were lysed with M-PER protein extraction reagent (Pierce, Rockford) supplemented with a protease inhibitor ‘cocktail’, then protein concentration in the extracts were measured by bicinchoninic acid assay (Pierce, Rockford). Equal amounts of extracts were separated by SDS-PAGE, and then were transferred onto PVDF membrane (Millipore) for immune-blot analysis as described before using anti-TAZ (Cell Signaling Technology, USA, #8418), β-actin (Santa Cruz, sc-1616-R).

### Reverse transcription PCR and quantitative real time PCR

Total RNA containing miRNA was extracted by Trizol (Invitrogen) from 6 × 10^5^ cells and quantified using a spectrophotometer (Eppendorf). 1 microgram of RNA was used for the reverse transcription into the cDNA using a Thermo Scientific RevertAid First Strand cDNA Synthesis kit. PCR was conducted according to the manufactures' instructions (Thermo Scientific). Target miRNA was reverse transcribed to cDNA by a gene-specific RT primer. miRNA expression profiles were determined with SYBR Green PCR kit (TIANGEN) and performed on BioRad Thermal Cycler. The relative quantification value of the target gene, was calculated by the comparative Ct methods. The primers used in the PCR were shown in Supplementary Table S2.

### Wound healing and transwell assay

Transfected cells grown in 24-well plate as confluent monolayer were scratched using a 10-μl pipette tip to create the wound. Cells were then washed with the culture medium to remove the cell debris and were cultured for another 24 hours to allow the wound healing. Transwell assay were performed without or with Matrigel (BD bioscience, migration or invasion respectively) coated on the upper surface of the transwell chamber (Corning, #3422). 12(migration) or 24(invasion) hours later, cells invaded through the transwell membrane were fixed by methanol and stained by crystal violet.

### *In vivo* tumor growth assay

6–8 week old male Balb/c mice (Experimental Animal Center, Shandong University) were bred in aseptic conditions. All mice were subcutaneously injected with mice hepatoma cell line H22 cells (2 × 10^6^) in left flank region. When visible tumor appeared, the mice were randomly divided into two groups. Tumors were injected with 20 μg of pCMV-miR-338-3p plasmid (Shanghai GenePharma Co., Ltd) or corresponding control plasmid in 100 transfection reagent (Polyplus-transfection, Inc, New York, USA) according to the manufacturer's instructions. The injection was performed once every 2 days for a total 14 days before the mice were sacrificed and the tumors were isolated. The tumor volume (V) was obtained by measuring the length (L) and width (W) with a caliper and calculated with the formula: volume (mm^3^) = L × W^2^/2. Mice were maintained in accordance with guidelines of the Institutional Animal Care and Use Committee, and all the animal studies were approved by Shandong University Institutional Animal Care and Use Committee.

### Statistical analysis

GraphPad Prism 5 (GraphPad Software, San Diego, CA) was used for data analysis. The student's *t*-test was applied to determined significant differences between groups. In these analyses, *p* value of less than 0.05 was considered statistically significant.

## SUPPLEMENTARY FIGURES AND TABLES


