# Supplementary material for: HBV preS2 promotes the expression of TAZ via miRNA-338-3p to enhance the tumorigenesis of hepatocellular carcinoma
Source: Oncotarget. 2015 Aug 5;6(30):29048–59. doi: 10.18632/oncotarget.4804 (PMC4745710; doi:10.18632/oncotarget.4804)
Supplement: Supplementary file 1 [file oncotarget-06-29048-s001.pdf]

SUPPLEMENTARY FIGURES AND TABLES

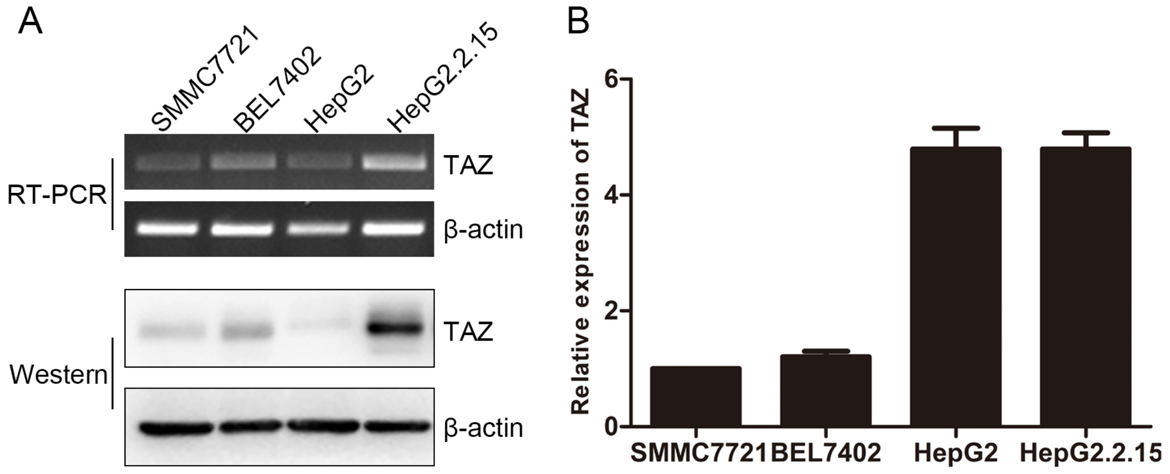

Supplementary Figure S1: Endogenous expression of TAZ in HCC cell lines. The endogenous expression of TAZ in HCC cell lines was detected by RT-PCR, Western Blot **A.** and qRT-PCR **B.** respectively.

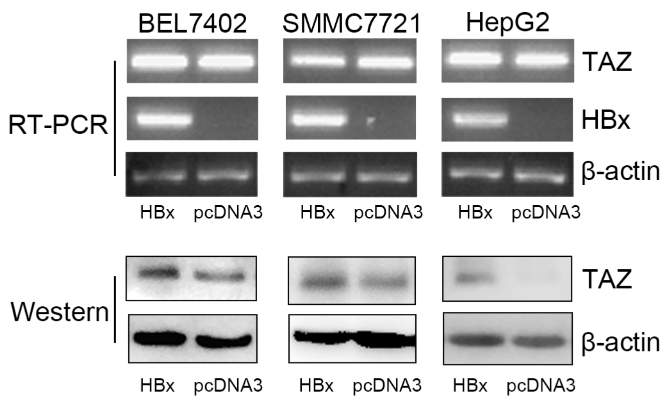

Supplementary Figure S2: HBx upregulates TAZ expression at the protein level. RT-PCR and western blot were used to detect TAZ expression in BEL7402, SMMC7721 and HepG2 cells transfected with pcDNA3 or HBx. β-actin served as the internal control.

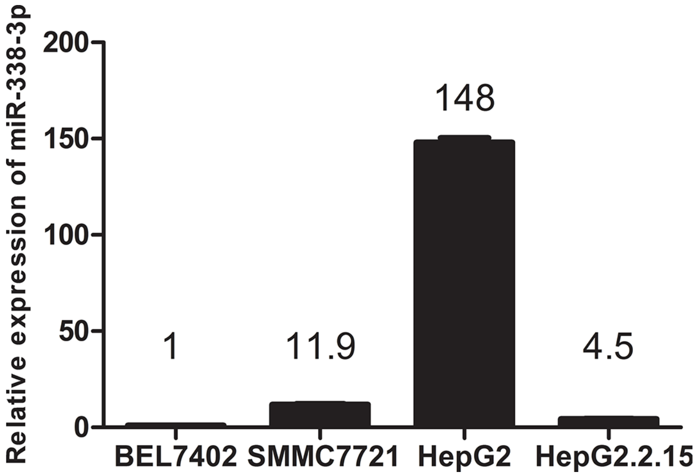

Supplementary Figure S3: Endogenous expression of miR-338-3p in HCC cell line. Endogenous expression of miR-338-3p in HCC cell lines was detected by qRT-PCR. U6 served as an internal control.

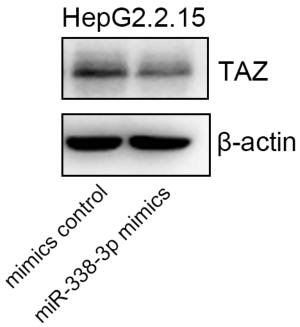

Supplementary Figure S4: miR-338-3p represses TAZ expression in HepG2.2.15. HepG2.2.15 cells were transfected with miR-338-3p mimics or mimics control and TAZ expression was analyzed by western blot. β-actin served as the internal control.

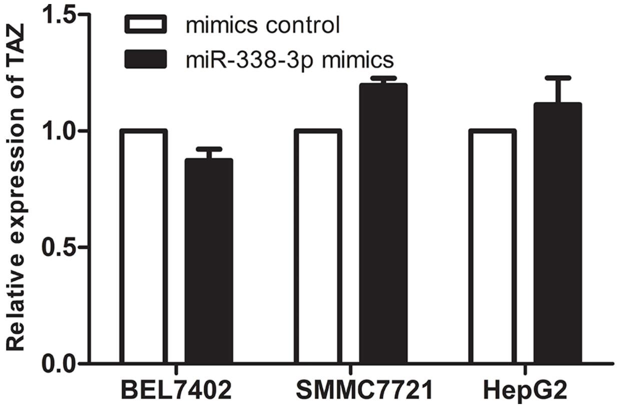

Supplementary Figure S5: Regulation of TAZ mRNA by miR-338-3p in HCC cell lines. miR-338-3p mimics was transfected into HCC cell lines and the expression of TAZ mRNA was detected by qRT-PCR. β-actin served as the internal control.

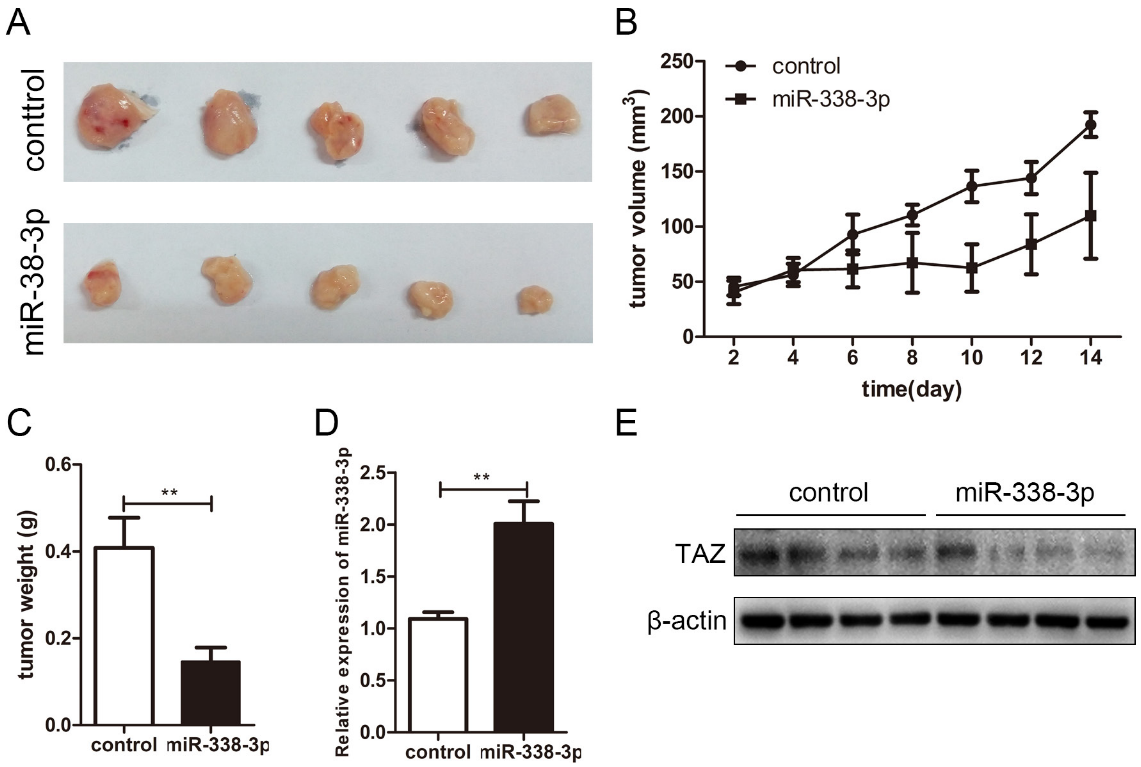

Supplementary Figure S6: miR-338-3p represses the growth of H22 homografts in Balb/c mice. A. The Balb/c mice were subcutaneously injected with H22 cells. Images presented were the isolated tumors transfected with miR-338-3p plasmid or control plasmid after sacrifice on day 14. **B.** The tumor volume was determined over a period of 14 days. **C.** Tumor weight was assayed on day 14 after the mice were sacrificed. **D.** Transfection efficiency was detected by qRT-PCR and U6 served as internal control. **E.** Expression of TAZ was determined by western blot. *$p < 0.05$, **$p < 0.01$.

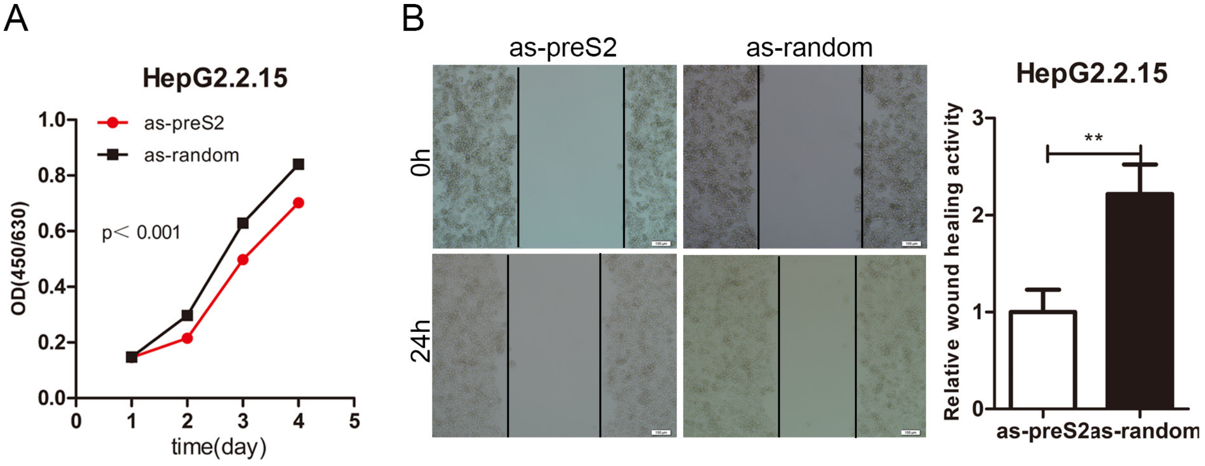

Supplementary Figure S7: Knockdown of preS2 impaired the proliferation and motility of HepG2.2.15. A, B. HepG2.2.15 was transfected with oligo as-random or as-preS2. The proliferation (A) and motility (B) was detected by CCK8 assay and wound healing assay respectively. **$p < 0.01$.

Supplementary Table S1: microRNAs potential targeting TAZ

	TargetScan	miRanda	PicTar
potential miRs	miR-338/338-3p	miR-222	miR-125b
	miR-141/200a	miR-221	miR-125a
	miR-144	miR-193b	miR-346
	miR-101/101ab	miR193a-3p	
	miR-490-3p	miR-544	
	miR-30abcdef	miR-129-5p	
	miR-30abe-5p	miR-320abcd	
	miR-384-5p	miR-1297	
	miR-29abcd	miR-26ab	
		miR-381	
		miR-300	
		miR-101	
		miR-411	
		miR-590-3p	

Supplementary Table S2: Primer sequence used in PCR

Gene	Primer sequence
U6	RT: CGCTTCACGAATTTGCGTGTCAT
	F: GCTTCGGCAGCACATATACTAAAAT
	R: CGCTTCACGAATTTGCGTGTCAT
miR-338-3p	RT: CTCAACTGGTGTCGTGGAGTCGGCAAT TCA GTT GAG CAA CAAAA
	F: ACA CTC CAG CTG GGT CCA GCA TCA GTG AT
	R: TGG TGT CGT GGA GTC G
miR-101	RT: GTCGTATCCAGTGCAGGGTCCGAGGTATTCGCACTGGATACGACTTCAGT
	F: GCGGCGGTACAGTACTGTGATAAC
	R: ATCCAGTGCAGGGTCCGAGG
miR-200a	RT: GTCGTATCCAGTGCAGGGTCCGAGGTATTCGCACTGGATACGACACATCG
	F: GCGGCGGTAACACTGTCTGGTAAC
	R: ATCCAGTGCAGGGTCCGAGG
miR-125a	RT: GTCGTATCCAGTGCAGGGTCCGAGGTATTCGCACTGGATACGACTCACAG
	F: GCGGCGGTCCCTGAGACCCTTT
	R: ATCCAGTGCAGGGTCCGAGG
miR-141	RT: GTCGTATCCAGTGCAGGGTCCGAGGTATTCGCACTGGATACGACTTCAGT
	F: GCGGCGGTAACACTGTCTGG
	R: ATCCAGTGCAGGGTCCGAGG
β-actin	F: CATGTACGTTGCTATCCAGGC
	R: CTCCTTAATGTCACGCACGAT
TAZ	F: GTCCTACGACGTGACCGAC
	R: CACGAGATTTGGCTGGGATAC
HBx	F: TCCTTTGTCTACGTCCCG
	R: TAATCTCCTCCCCCAACTCCTC
preS2	F: CCACCATGCAGTGGAACTC
	R: TGTGTTCTCCATGTTCGGTG

Supplementary Table S3: siRNA sequence used in the experiment

Gene	Sequence
TAZ siRNA1	Sense: 5′ GGUACUUCCUCAAUCACAUT3′
TAZ siRNA2	Sense: 5′ GCUCAUGAGUAUGCCCAAUTT3′
TAZ siRNA3	Sense: 5′ CCGUUUCCCUGAUUUCCUUTT3′
